# Genomic Signature-Based Identification of Influenza A Viruses Using RT-PCR/Electro-Spray Ionization Mass Spectrometry (ESI-MS) Technology

**DOI:** 10.1371/journal.pone.0013293

**Published:** 2010-10-12

**Authors:** Varough M. Deyde, Rangarajan Sampath, Rebecca J. Garten, Patrick J. Blair, Christopher A. Myers, Christian Massire, Heather Matthews, Pavel Svoboda, Matthew S. Reed, Jan Pohl, Alexander I. Klimov, Larisa V. Gubareva

**Affiliations:** 1 Influenza Division, National Center for Immunization and Respiratory Diseases, Centers for Disease Control and Prevention, Atlanta, Georgia, United States of America; 2 Genomics & Computational Biology, Ibis Biosciences, Carlsbad, California, United States of America; 3 Naval Respiratory Disease Laboratory, Naval Health Research Center, San Diego, California, United States of America; 4 Division of Scientific Resources, National Center for Emerging and Zoonotic Infectious Diseases, Centers for Disease Control and Prevention, Atlanta, Georgia, United States of America; Singapore Immunology Network, Singapore

## Abstract

**Background:**

The emergence and rapid spread of the 2009 H1N1 pandemic influenza A virus (H1N1pdm) in humans highlights the importance of enhancing the capability of existing influenza surveillance systems with tools for rapid identification of emerging and re-emerging viruses. One of the new approaches is the RT-PCR electrospray ionization mass spectrometry (RT-PCR/ESI-MS) technology, which is based on analysis of base composition (BC) of RT-PCR amplicons from influenza “core” genes. Combination of the BC signatures represents a “genomic print” of an influenza A virus.

**Methodology/Principal Findings:**

Here, 757 samples collected between 2006 and 2009 were tested, including 302 seasonal H1N1, 171 H3N2, 7 swine triple reassortants, and 277 H1N1pdm viruses. Of the 277 H1N1pdm samples, 209 were clinical specimens (throat, nasal and nasopharyngeal swabs, nasal washes, blood and sputum). BC signatures for the clinical specimen from one of the first cases of the 2009 pandemic, A/California/04/2009, confirmed it as an unusual, previously unrecognized influenza A virus, with “core” genes related to viruses of avian, human and swine origins. Subsequent analysis of additional 276 H1N1pdm samples revealed that they shared the genomic print of A/California/04/2009, which differed from those of North American swine triple reassortant viruses, seasonal H1N1 and H3N2 and other viruses tested. Moreover, this assay allowed distinction between “core” genes of co-circulating groups of seasonal H1N1, such as clades 2B, 2C, and their reassortants with dual antiviral resistance to adamantanes and oseltamivir.

**Conclusions/Significance:**

The RT-PCR/ESI-MS assay is a broad range influenza identification tool that can be used directly on clinical specimens for rapid and accurate detection of influenza virus genes. The assay differentiates the H1N1pdm from seasonal and other nonhuman hosts viruses. Although not a diagnostic tool, this assay demonstrates its usefulness and robustness in influenza virus surveillance and detection of novel and unusual viruses with previously unseen genomic prints.

## Introduction

Influenza A viruses are important respiratory pathogens that cause annual epidemics and occasional pandemics. The 2009 H1N1 pandemic (H1N1pdm) is a reminder of the need to develop and improve tools for rapid identification of emerging and novel viruses. Influenza A viruses consist of a segmented negative sense RNA genome which is prone to acquisition of point mutations and gene reassortment [1]. The two major surface antigens, the hemagglutinin (HA) and neuraminidase (NA) glycoproteins, are coded by their respective HA and NA genes. The remaining six “core” genes (PB1, PB2, PA, NP, M, and NS) encode eight to nine viral proteins (PB1, PB1-F2, PB2, PA, NP, M1, M2, NS1, and NS2) [2], [3].

Influenza A virus evolution is considered to be predominantly driven by host-mediated selective pressure that leads to amino acid replacements at key antigenic sites in the HA, a mechanism known as antigenic drift [4], [5]. Another mechanism of influenza virus evolution, antigenic shift, which occurs at a lower frequency, involves the replacement of HA and/or NA with new antigenic virus subtypes that have not circulated in humans viruses for a long time [6].

Inter-subtype reassortment is rare, whereas intra-subtype reassortment occurs more often among distinct co-circulating lineages of the same influenza A virus subtype [7], [8]. In particular, intra-subtype reassortment events have been reported to result in the acquisition of drug resistance and may facilitate the spread of drug resistant viruses [9], [10]–[12]. In addition to natural reassortment, live attenuated vaccines are generated to include HA and NA genes from epidemiologically relevant strains and the “core genes” from the master donor viruses (e.g. cold adapted A/California/7/09(H1N1pdm) x A/Ann Arbor/6/60(H2N2)) [13].

In April 2009, a previously unseen virus emerged and rapidly spread globally leading to the first influenza pandemic of the 21^st^ century [14]. The H1N1pdm virus has a complex genome composition, with genes originating from swine, human and avian influenza A viruses. Complete genome sequencing and phylogenetic analysis demonstrated that the virus had genes related to North American triple reassortant and Eurasian swine viruses [15]–[17], including the M gene of adamantane-resistant Eurasian avian-like swine viruses [16].

The continuous evolution of influenza genomes together with reassortment events pose challenges to the effective monitoring of influenza viruses in circulation. Current methods of laboratory influenza surveillance are primarily based on hemagglutinin inhibition (HI) and other assays utilizing the antibody recognition of viral antigens [18], [19]; conventional or real-time RT-PCR methods [20]–[24]; and sequencing coupled with phylogenetic analysis (e.g. as in [16]). These methods, though commonly used, each has certain unique advantages but also some limitations. For example, some are designed for rapid testing, without greater attention given to the degree of sensitivity or specificity; others allow virus typing but do not provide details on subtypes; and some of the more sensitive and accurate methods may not but suited high throughput testing …etc.

Oftentimes, there is a strong correlation between the sequences of the “core” genes and the “surface” HA and NA genes of influenza A viruses [25]. Thus, in the absence of a reassortment, identification of “core” genes can be used to infer the viral HA and NA subtypes. Noteworthy is the fact that reassortment is rather common among porcine viruses (e.g., H1N2, H3N1, H1N1, and H3N2) [26], thus making it more challenging to infer their subtypes based on core gene constellation.

The RT-PCR/ESI-MS assay was previously introduced for detection and characterization of influenza viruses [25], [27], [28]. The BC signatures of each amplicon are then determined using automated ESI-MS signal acquisition and spectral analysis.

Identification of each influenza A virus is based on the summation of BC signatures obtained from the six target genes (PB1, NP, M1, PA, NS1, and NS2). This combination of BC signatures is referred to as “genomic print” throughout the manuscript, and is used for comparison to previously known viruses with BC signatures and/or genomic prints in a reference database. The viruses are identified by the virus(es) in the database with the closest match to their genomic prints/or BC signatures. For instance, if a virus has a genomic print identical or similar to a known reference virus, it will be identified as such. On the other hand, if a virus has a genomic print that is not identical or similar to a single reference virus (e.g. different genes from different subtypes or different hosts); it will be identified with more than one virus (e.g. in the case of a reassortant). Finally, if the virus has no known close matches to its BC signatures in the database, it will be identified as “unknown”.

In the present study, we focused on the influenza RT-PCR/ESI-MS assay's ability to identify influenza A viruses affecting humans. Based on our previous experience with the assay using seasonal influenza viruses, and its reported performance in analysis of influenza A(H3N2) [25], the main goals of this study were to investigate: (**A**) whether the RT-PCR/ESI-MS assay can be applied for identification of the H1N1pdm viruses; (**B**) whether this method could be helpful in the analysis of A(H3N2) virus lineages from the 2006-07 to 2008-09 seasons; (**C**) whether the assay could identify and distinguish between the major lineages of seasonal H1N1 viruses (2B and 2C clades), including the dual oseltamivir and adamantane-resistant viruses; and lastly (**D**) whether wild type viruses could be readily distinguished from the live attenuated vaccines (LAIV) using this assay.

Our efforts show that that the RT-PCR/ESI-MS assay provides a broad range, high throughput identification tool that could be used for influenza virus surveillance purposes. It can successfully detect novel viruses, including those arising from the reassortment of ‘core’ genes, and the assay has the ability to differentiate among genetic groups (lineages or clades) within the same virus subtype.

## Results

### Identification of the H1N1pdm viruses using RT-PCR/ESI-MS

**Figure 1:**
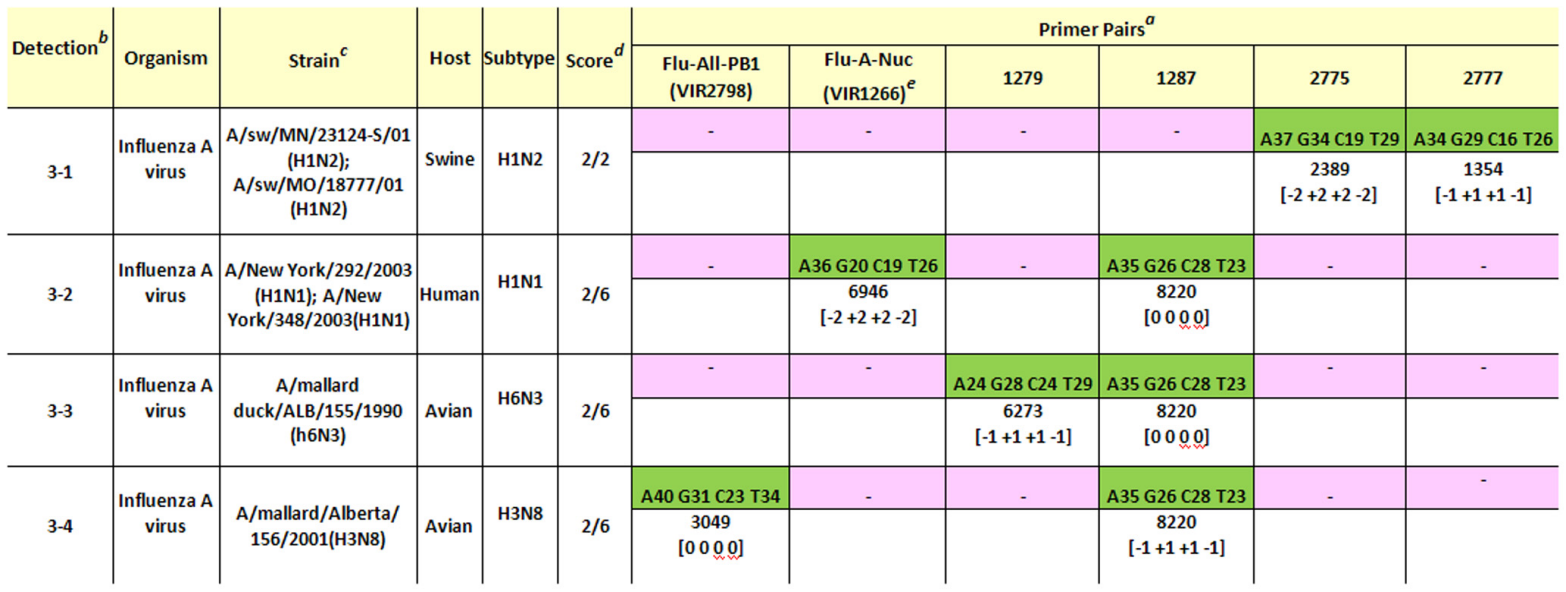
RT-PCR/ESI-MS identified initial H1N1pdm strain as unusual virus with genome components of swine, human, and avian origin. RNA from clinical specimens was prepared from one of the first cases of the 2009 pandemic and tested using the RT-PCR/ESI-MS assay. BC signatures did not match any of the known reference genomic signatures in the database. The signatures indicated a close relationship to swine, human and avian sequences. The gene segments targeted were PB1 (VIR2798), NP (VIR1266), M1 (1279), PA (1287), NS1 (2775), and NS2 (2777). The base counts determined for each target are highlighted in green; below them are the number of viral RNA copies and the SNP variations from the matching strain, respectively. *^a^* VIR2798 =  PB1; VIR1266 =  NP; 1279 =  M1; 1287 =  PA; 2775 =  NS1; 2777 =  NS2; *^b^* The tested sample had 4 different close matches in the reference database, depending on the six targets; *^c^* The closest matching virus, from reference the database, to the sample tested; *^d^* The numerators refer to # of targets identified. The denominators refer to the number of entries (# of genes) from the different viruses represented with BC signatures in the database; *^e^* The sample tested had matches to viruses from swine, human, and avian, but identification of origin was not correct for all genes (e.g. the NP had a closest match in a human H1N1 virus, while genome sequencing revealed the virus has NP related to North American triple reassortant swine H1N1).

**Figure 2:**
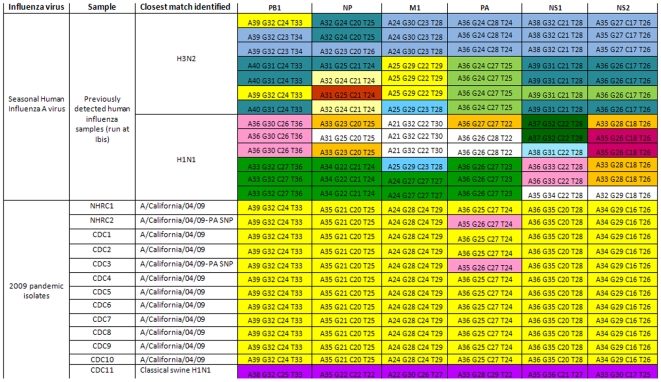
The genomic print of H1N1 pdm viruses is highly conserved and distinct from other influenza viruses. Thirteen (13) seasonal H1N1 and H3N2 viruses from various seasons (top half); 12 samples from the H1N1pdm viruses (including the two viruses of the first two cases of the 2009 pandemic, referred to as NHRC1 and 2) (bottom half in yellow highlight); and a North American swine of H1N1 subtype were analyzed. All 2009 H1N1 pdm viruses have the same genomic print. BC signatures of five of the six targets in the H1N1pdm virus (NP, M1, PA, NS1, and NS2) were different from the other subtypes. The BC signature determined for the PB1 (A39, G32, C24, T33) was identical to some human H3N2 viruses, as expected. The H1N1pdm viruses were also distinguishable from the triple reassortant swine H1N1 at all six targets (compare yellow and purple signature). Differences in color patterns within the same subtype either reflects different genetic groups (H3N2 and H1N1), or a single nucleotide polymorphism (2009 H1N1 pdm). The numbers preceded by letters in each box correspond to the base counts (number A, G, C, and T) determined from the amplicons of the respective target genes.

The RT-PCR/ESI-MS assay was applied to test the “unsubtypable” clinical specimen of the first US H1N1pdm virus, A/California/4/2009, at the Naval Health Research Center (NHRC), in San Diego CA, USA [29]. The results (Figure 1) showed no match to any known genomic prints in the database, but clearly indicated the presence of an unusual influenza A virus, showing BC signatures of individual genes that were similar to those from viruses of swine, human, and avian origins. It should be noted that although this initial identification showed deficiencies in accurately determining the origin of each gene of this complex reassortant virus, results such as these would alert the investigator of the need for further analysis through the use of traditional methods. After the complete genome sequence and phylogenetic analysis of the A/California/4/2009 were examined [16], the BC signatures of this virus were added to the database. Its genomic print was easily distinguishable from those of seasonal H1N1 and H3N2 and other influenza A viruses (Figure 2), and became the reference for the identification of subsequent H1N1pdm viruses. It is also important to note that the BC signatures of the pandemic virus are readily discernible from those of swine triple reassortant viruses that have previously caused infections in humans [15]. The criterion used for future identification of the H1N1pdm viruses was based on the detection of BC signatures identical to those of A/California/04/2009 in at least five of the six targets in the “core” genes. The original screen shot obtained directly from the T5000 instrument (Figure 1) is presented as Figure S1.

### Use of RT-PCR/ESI-MS for high throughput screening and monitoring of the H1N1pdm viruses for genomic variations

To further assess the ability of the RT-PCR/ESI-MS assay to accurately identify the pandemic viruses and determine its sensitivity and specificity, we tested 401 specimens previously characterized using the CDC rRT-PCR assay [http://www.who.int/csr/resources/publications/swineflu/CDCrealtimeRTPCRprotocol_20090428.pdf]. These samples were collected during the early stages of the pandemic, between April and June, 2009. One hundred forty-four (144) of them were identified as H1N1pdm using the RT-PCR/ESI-MS; while based on CDC-rRT-PCR assay results, 152 of them were positive for the H1N1pdm. One sample tested positive in the RT-PCR-ESI-MS but negative in the rRT-PCR. This corresponds to an overall sensitivity of ∼94.1% (Table 1). Among these positive samples, 120 were from residual RNA from clinical specimens and the remaining were grown viruses. Of the 249 specimens identified as negative rRT-PCR, 248 also tested negative by RT-PCR/ESI-MS, corresponding to a specificity of 99.59% (Table 1). Four of the 9 samples that were identified as positive based on the rRT-PCR but negative by the RT-PCR/ESI-MS matched the reference H1N1pdm virus signatures in less than five targets, which is below the acceptance criteria, set in the system. The remaining five samples had less than adequate volume of RNA material. Of note, after the sensitivity and specificity analyses were performed on the early specimens and more samples became available, another set of 133 left over RNAs of confirmed cases of H1N1pdm by the rRT-PCR (including 89 from clinical specimens) were analyzed in the RT-PCR/ESI-MS assay and were confirmed as such. This brought the final total number of H1N1pdm viruses identified by this assay to 277, with 209 being from original clinical specimen.

**Table 1:** Sensitivity and specificity of the RT-PCR/ESI-MS assay in detecting the 2009 H1N1pdm in comparison to the CDC rRT-PCR assay.

		CDC Real time RT-PCR Assay		
		Positive	Negative	Total
	Positive	143	1	144
**T5000**	Negative	9	248	257
	Total	152	249	401
		Sensitivity	94.1	
		Specificity	99.6	
		Pos Pred Value	99.3	
		Neg Pred Value	96.5	

During the period from April to June, a total of 401 RNA samples randomly collected during the early stages of the pandemic were tested in the assay. One hundred forty-four (144) of them were identified as H1N1pdm using the RT-PCR/ESI-MS; while based on CDC-rRT-PCR assay results, 152 of them were positive for the H1N1pdm. This corresponds to a sensitivity of 94.07%. Among these positive samples, 120 were from residual RNA from clinical specimens and the remaining were grown viruses. Of the 249 specimens identified as negative rRT-PCR, 248 also tested negative by RT-PCR/ESI-MS, corresponding to a specificity of 99.59%. Four of the 9 samples that were identified as positive based on the rRT-PCR but negative by the RT-PCR/ESI-MS matched the reference H1N1pdm virus signatures in less than five targets, which is below the acceptance criteria, set in the system. The remaining five samples had less than adequate volume of RNA material. Of note, after the sensitivity and specificity analyses were performed, and more samples became available, another set of 133 left over RNAs of confirmed cases of H1N1pdm (including 89 from clinical specimens) were analyzed in the RT-PCR/ESI-MS assay and were confirmed as such. This brought the final total of H1N1pdm viruses identified by this assay to 277, with 209 being from original clinical specimen.

As shown in Figure 2, BC signatures of the H1N1pdm viruses were clearly distinguishable from those of seasonal H1N1, H3N2, as well as from swine triple reassortant H1N1 viruses. BC signature patterns were color-coded for clarity. The genomic prints of most samples tested were identical and consistent with that of the reference virus A/California/04/2009. However, approximately 10% of the samples showed a single nucleotide polymorphism (SNP) in the PA gene at nucleotide 618 (A to G mutation) (Figure 2). The base counts results obtained for each amplicon, determined by the RT-PCR/ESI-MS, were compared and found consistent with the genome sequencing data available for a subset of the viruses analyzed here [16].

**Figure 3:**
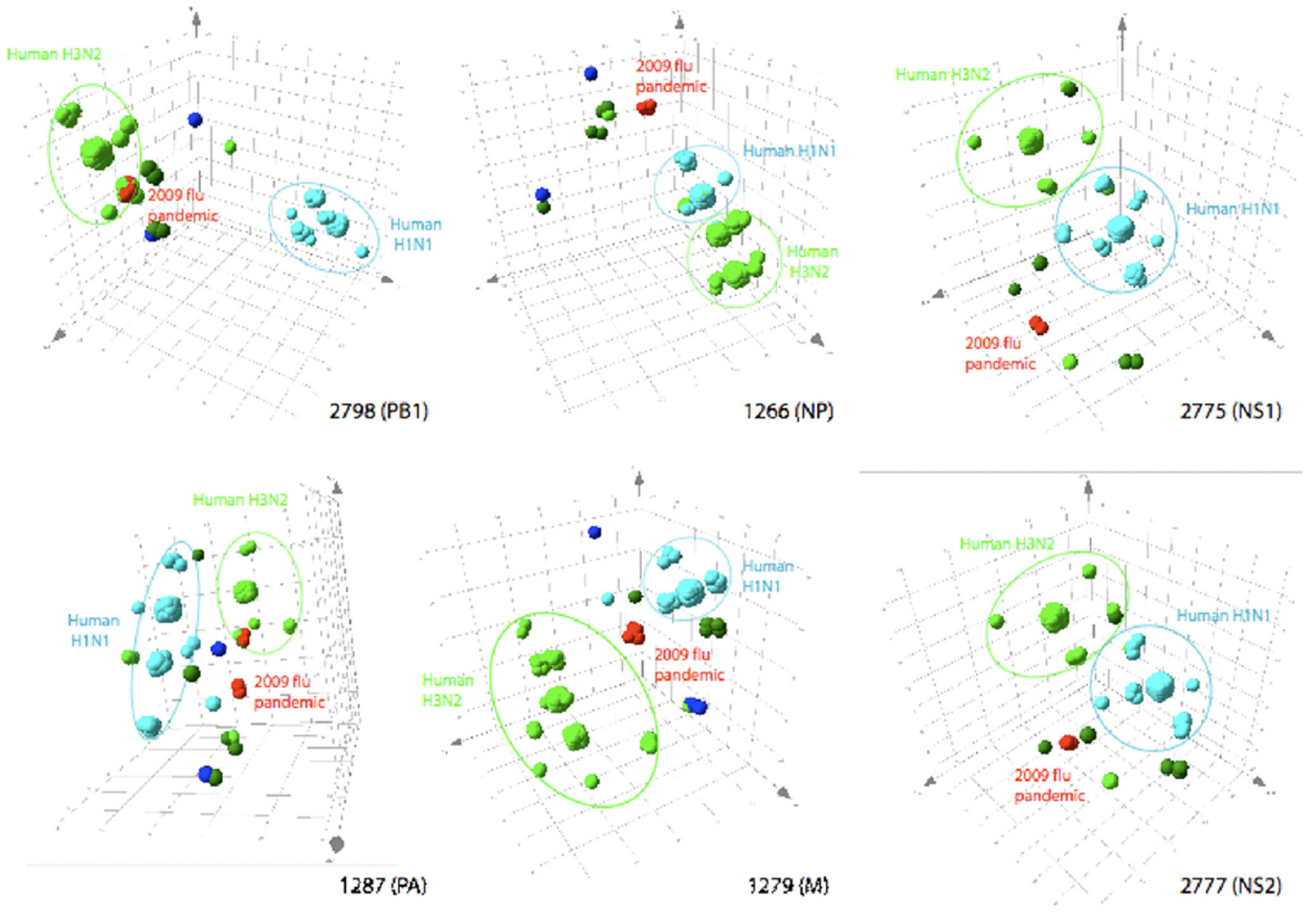
Spatial representation of BC signatures covered by the six primer pairs for Influenza A viruses characterization. Black numerals refer to the individual primer pair numbers, and are followed by the designation of the segment from which they stem (between parentheses). For each primer pair, each sphere represents one strain of influenza circulating within the seasons 2007–2009, and is color coded by host and subtype: 1) light blue, human H1N1; 2) dark blue, swine triple reassortant H1N1; 3) light green, human H3N2; 4) dark green, swine triple reassortant H3N2; 5) and red, H1N1pdm. For clarity, ellipses regroup the most common subtypes in circulation. Each isolate is mapped in a three-dimensional space according to its amplicon BC signature using the designated primer pair.

Distinction between the BC signatures of the H1N1pdm virus from those of other stable influenza A subtype viruses collected between 2006-07 and 2008-09 (Table S1), is further illustrated in Figure 3. Details on the spatial representation of the BC signature data is described elsewhere [25]. Superposition of BC from the H1N1pdm viruses showed that for five of the six target regions, the H1N1pdm signatures are unique. The PB1 signatures of H1N1pdm and human H3N2 viruses overlapped as expected based on phylogenetic analysis of this gene segment [16].

Results from the limits of the detection studies of the H1N1pdm virus using the RT-PCR/ESI-MS assay showed that the lowest detectable concentrations of the virus varied from 7.0×10^1^ TCID_50_/ml to 2.11×10^2^ TCID_50_/ml, depending on the matrix (nasal swab or nasal wash) used for sample dilution (Table S2). Analysis of sensitivity of the assay based on viral genome copy numbers revealed that the LoD of the assay was 31 and 62 genome copies, from the nasal swab and nasal wash, respectively, using the Ambion MagMax® Viral RNA Isolation kit. The same analysis using the Qiagen QIAamp® MinElute Virus Spin kit demonstrated that the assay's LoD of was 125 and 62 viral genome copy numbers, from the nasal swab and nasal wash, respectively. Of note, the limit of detection here (the numbers) is considered only if all primers generated PCR products and BC signatures in the assay (Figure S2).

### Analysis of seasonal A(H3N2) viruses circulating from 2006-07 to 2008-09 seasons using RT-PCR/ESI-MS

During the 2006-07 season, new antigenic variants of A(H3N2) viruses emerged and were different from the vaccine strain A/Wisconsin/67/2005. In addition, distinct patterns of adamantane susceptibility were observed among the co-circulating genetic groups. These initial findings prompted us to evaluate the usefulness of the RT-PCR/ESI-MS method in screening a large number of H3N2 viruses in order to identify major genetic lineages co-circulating in different parts of the world. Such information could be useful in making decisions and recommendations on new vaccine strain selection and M2 antiviral drug (adamantanes) usage. To this end, 76 viruses collected from various geographic regions in 2006–2007 were analyzed. Twenty unique genomic prints were identified. The most dominant genomic print among the adamantane-resistant viruses (AADFAA) was shared with H3N2 viruses that circulated in prior seasons (N-lineage) [25]. Table 2 represents the identified genomic prints. In parallel to the RT-PCR/ESI-MS, full genome sequencing and phylogenetic analyses were performed on 65 of the 76 viruses [12]. Comparison of results from both approaches showed that the genomic prints identified by the RT-PCR/ESI-MS belong mainly to one of the four major genetic groups determined by phylogenetic analysis: 1) two adamantane-sensitive groups, A/Nepal/921/2006-like and A/Brisbane/09/2006-like, and 2) two adamantane-resistant groups, A/Nepal/921/2006-N144D-like and A/Brisbane/10/2007-like (Table 2). Two genomic prints were identified for 2 viruses belonging to the N-lineage; while the three remaining genomic prints were unique to three viruses which circulated prior to the N-lineage appearance.

**Table 2:** Identification of unique genomic prints in H3N2 viruses circulating during the 2006-07 influenza season and their correlation with genome sequencing and M2 susceptibility data.

Strain Designation	Genomic prints†	Genome sequencing lineages	M2- blockers susceptibility*
A/Wisconsin/02/2007	AEEFAA	B- (Nepal/921/2006-like)	S
A/Nepal/921/2006	CEEFAA	B- (Nepal/921/2006-like)	S
A/Bangladesh/1999/2006	GEBFAA	B- (Nepal/921/2006-like)	S
A/Indiana/03/2007	CDEFAA	B- (Nepal/921/2006-like)	S
A/Lyon/10/2006	CEAFAA	B- (Nepal/921/2006-like)	S
A/Korea/7212/2007	AADFAA	A- (Nepal/921/2006-N144D-like)	R
A/Korea/7210/2007	AABFAA	A- (Nepal/921/2006-N144D-like)	R
A/Japan/7288/2007	CADFAA	A- (Nepal/921/2006-N144D-like)	R
A/Incheon/701/2006	ACDFAA	A- (Nepal/921/2006-N144D-like)	R
A/Wisconsin/43/2006	AEJBAA	C-(Brisbane/09/2006-like)	S
A/Bordeaux/1276/2006	ECJBAA	C-(Brisbane/09/2006-like)	S
A/Lyon/CHU/52.58/2006	CCJBAA	C-(Brisbane/09/2006-like)	S
A/Daejeon/690/2006	ACJFAA	C-(Brisbane/09/2006-like)	S
A/New Mexico/02/2007	CCLFAA	D-(Brisbane/10/2007-like)	R
A/Idaho/03/2007	CCDFAA	D-(Brisbane/10/2007-like)	R
A/Kentucky/3e/2006	CEDBAA	N-lineage	R
A/Venezuela/6971e/2005	ACBBAA	N-lineage	R
A/Omsk/32/2004‡	AABNAA	Pre-N-lineage	R
A/Fujian/411/2002‡	HADFAA	Pre-N-lineage	S
A/Wuhan/396/2001‡	AADBAA	Pre-N-lineage	R

The six letters in the genomic prints for each virus refer to different BC signatures determined based on the six targets. (e.g. A/Wisconsin/02/2007 has BC signature for PB1 that was designated A, BC signatures at NP and M1 were each designated E, its BC signature at PA was named F, and the BC signatures at NS1, and NS2, were each designated A).

‡ Viruses were collected prior to 2005 and were used as reference viruses.

*S: sensitive; R: resistant.

RT-PCR/ESI-MS analysis of 95 H3N2 viruses, collected during 2007-08 and 2008-09 influenza seasons (57 and 38, respectively, Table S1), revealed that all shared the genomic print of the reference virus A/Brisbane/10/2007 and no evidence of major genetic changes were detected based on the “core” gene segments analyzed.

Together, these findings show that the assay could be used as a primary screening tool to identify major genetic groups based on their genomic prints. This can help in reducing the redundancy of sequencing identical viruses by identifying those with unique genetic features and not duplicating the same sequences.

### Identification of genetic groups of co-circulating seasonal A(H1N1) viruses

The emergence and co-circulation of seasonal pre-pandemic H1N1 viruses resistant to oseltamivir and/or adamantanes has been a serious public health concern. Recently, an increasing number of dually resistant viruses that emerged from reassortment between two genetic lineages (clades 2B and 2C) of seasonal H1N1 viruses was reported [30], [31], [32]. We assessed the ability of the RT-PCR/ESI-MS assay to identify such reassortants.

**Figure 4:**
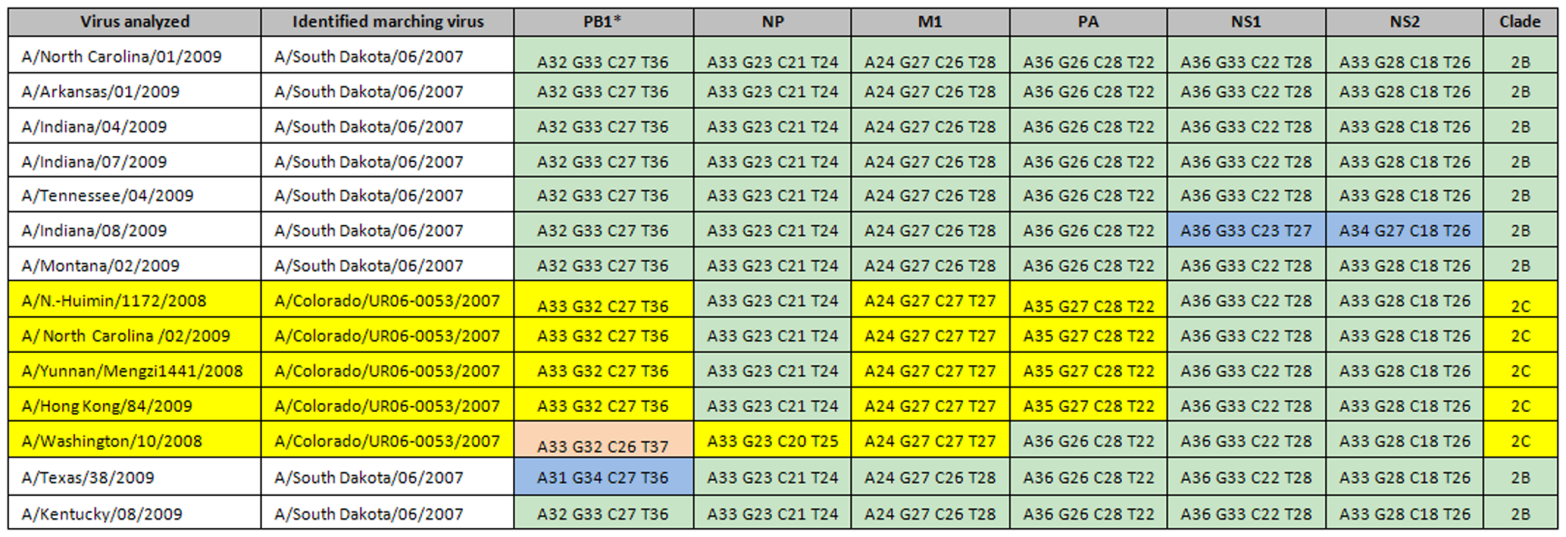
RT-PCR/ESI-MS assay allows differentiation between the two major seasonal H1N1virus clades 2B and 2C. Fourteen H1N1 viruses with previously known clades (based on phylogenetic data) were blindly tested and the results were analyzed against data base reference viruses: A/South Dakota/06/2007 and A/Colorado/UR06-0053/2007, of clades 2B and 2C, respectively. Nine of the viruses were identified as clade 2B and the remaining five as clade 2C, mainly based on the combination of BC signatures of PB1, M1, and PA (highlighted in yellow). The remaining 3 targets (NP, NS1, and NS2) had identical BC signatures in both groups of viruses (highlighted in light green). One or two SNPs were also detected in two viruses (light blue) but had no effect on clade identification. *The numbers preceded by letters in each box correspond to the base counts (number A, G, C, and T) determined from the amplicons of the respective target genes.

First, 14 seasonal H1N1 samples with known full genome phylogenies were tested to identify the clades to which they belonged based on the RT-PCR/ESI-MS. This assay successfully identified viruses from clades 2B (n = 9) and 2C (n = 5) (Figure 4). The genomic prints of these two seasonal H1N1 groups differed from each other mainly by the PB1, PA, and M1 genes, with M1 gene providing the highest dissimilarity resolution.

**Figure 5:**
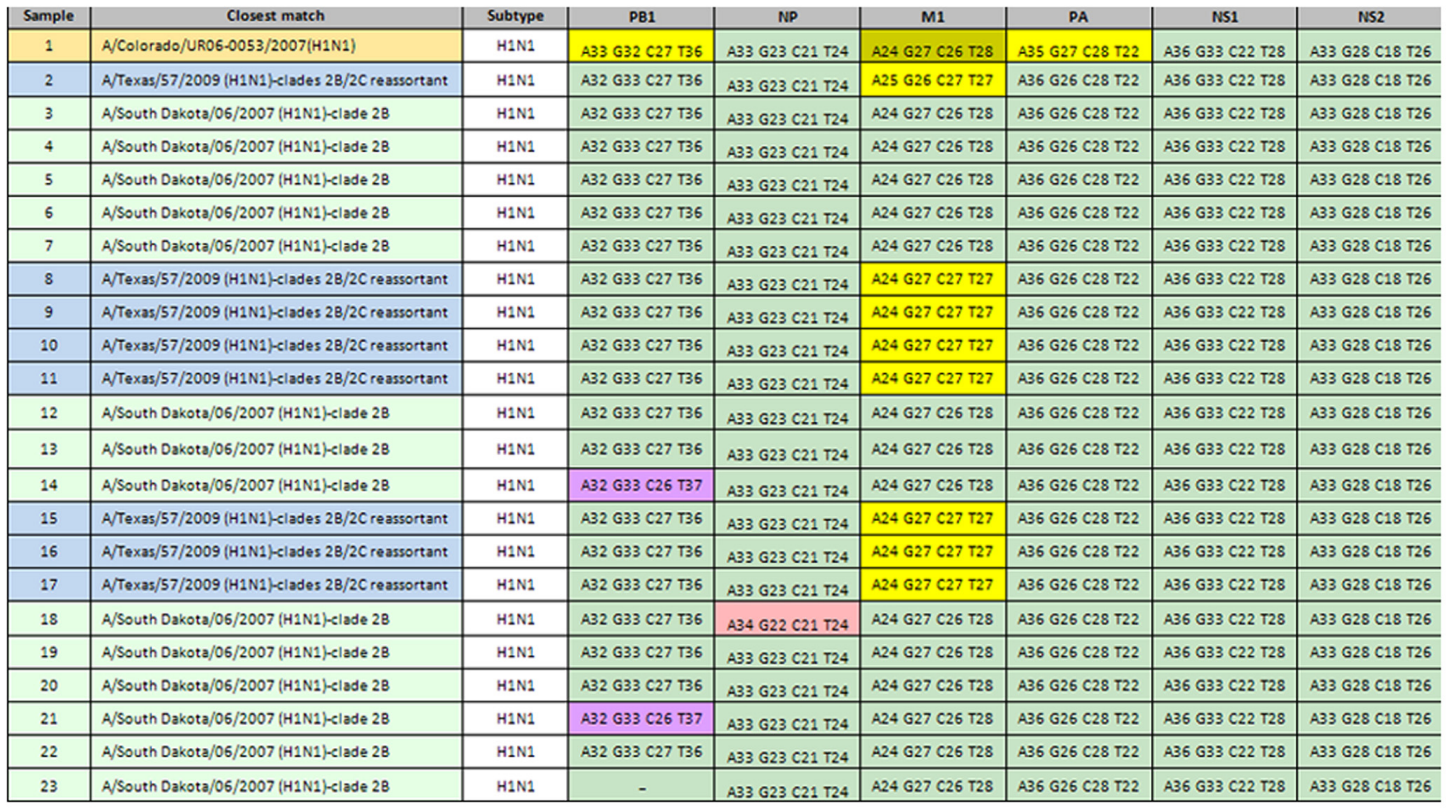
Detection of intra-subtype reassortment among seasonal H1N1 viruses using the RT-PCR/ESI-MS assy. A set of 23 viruses with unusual drug resistance profiles (resistant to both oseltamivir and the adamantanes) were analyzed; fourteen (14) of them showed genomic prints identical to the prototype clade 2B virus, A/South Dakota/06/2007 (green highlight), based on all six targets, with the exception of three viruses that had single SNPs each in the PB1 amplicon (two) and the NP (one) (light purple). Sample 23 did not amplify with the PB1 target. Seven viruses had five BC signatures (from PB1, NP, PA, NS1, and NS2) matching the clade 2B virus, while their M1 BC signature was typical of clade 2C virus, A/Colorado/UR06-0053/2007 (light yellow). Two viruses had genomic prints uncharacteristic of either clade 2B or clade 2C (samples 1 and 2). The numbers preceded by letters in each box correspond to the base counts (number A, G, C, and T) determined from the amplicons of the respective target genes.

Next, 23 viruses with known HA, NA, and M gene segments phylogenies were tested. Fourteen had genomic prints identical to that of the clade 2B reference virus, A/South Dakota/06/2007, with two viruses each having one SNP in the PB1 amplicon, one with a SNP in the NP gene target, and one that failed to amplify using the PB1 primers (Figure 5). Seven viruses had hybrid BC signatures from both clades 2B and 2C: the PB1, NP, PA, NS1, and N2 BC signatures belonged to clade 2B, while the M1 BC signature was clearly that of clade 2C, suggesting acquisition of the clade 2C M segment by viruses from clade 2B by reassortment. The data were similar to the findings of the phylogenetic analysis: these viruses with hybrid BC signatures matched the genomic print of A/Texas/57/2009, a known reassortant virus with all genes from clade 2B, except the M gene, which is acquired by reassortment from a clade 2C virus [32]. The remaining two viruses (Figure 5, samples 1 and 2) contained SNPs in their M1 genes and thus, the identification of the clades to which they belong could not be confirmed.

Noteworthy, the genomic prints of seasonal H1N1 viruses including the identified reassortants were distinguishable from other influenza A viruses.

### Distinction between live attenuated influenza vaccine (LAIV) and the wild type 2009 H1N1 pdm viruses using the RT-PCR/ESI-MS

Since live attenuated vaccines contain the ‘core genes’ of the master donor virus A/Ann Arbor/6/60 (H2N2), it was essential to investigate if its genomic print significantly differs from those of wild type viruses in circulation. Indeed, the identified genomic print of the H1N1pdm LAIV was clearly distinguishable from that of wild type 2009 H1N1 pdm virus, seasonal H1N1, seasonal H3N2 viruses and other viruses of animal origin. Figure S3 represents results of RT-PCR/ESI-MS analysis of an H1N1pdm LAIV case (ID: 2010703400).

The results indicate that the assay facilitates distinction between the wild type 2009 H1N1 pdm and its cold adapted vaccine strain.

## Discussion

Pandemic influenza viruses have caused morbidity and mortality in humans and have negatively impacted the world's economy [33]. The availability of tools that allow rapid identification of novel viruses during routine surveillance is critical in pandemic preparedness and response management.

The present study describes the use of the RT-PCR/ESI-MS assay at the Naval Health Research Center (NHRC) to identify the “unsubtypable” influenza A virus present in the specimen from one of the first cases of H1N1pdm [17], [29]. The results generated at NHRC using the standard RT-PCR/ESI-MS Influenza assay indicated that the virus was different from the currently known circulating human influenza viruses. This new virus had distinct BC signatures that suggested genome contributions from swine, human and avian influenza A viruses. Nevertheless, its accurate identification as a swine-origin reassortment of H1N1 subtype was made only after genome sequence and phylogenetic analyses were performed at CDC and other laboratories [16], [34]. This same Influenza RT-PCR/ESI-MS assay has been used at the CDC since 2007 to test seasonal viruses, and was also independently used for identification of the H1N1pdm viruses soon after characterization of the first isolates.

The RT-PCR/ESI-MS assay, despite being capable of identifying novel and emerging viruses, is not an alternative to conventional virus characterization methods such as rRT-PCR and complete genome sequencing complemented with phylogenetic analysis. However, the RT-PCR/ESI-MS occupies its own niche by determining the genomic prints of “core” genes of viruses analyzed and rapidly providing a snap shot of their genome composition on the basis of previously characterized standards. This assay allows testing of ∼300 samples in 24 h, with first results obtained within 6 h [28]. In its current form, the assay is semi-automated, with sample preparation and amplifications performed outside the system, whilst all post-PCR amplification steps are automated in the T5000 biosensor. Recently, numerous high throughput sequencing technologies have been developed to allow rapid and cost-effective ways to obtain genome-wide information [35]–[37]. However, it is important to underline that choosing which of these methods to use is often driven by a combination of a number of factors including: sensitivity, specificity, fitness-for-purpose, technical ease, and time and cost of effectiveness. The influenza RT-PCR/ESI-MS assay lends itself as a complementing tool to the array of assays currently in use, especially in the field of surveillance; and like any other technology, it has its own advantages and limitations. For example, this assay has some similarities with Sequenom MassARRAY iPLEX genotyping platform [38]. Both are based on measurement of masses of nucleotides from an amplified target. However, they differ in that the latter is aimed determining a single SNP per amplicon. This assay allows multiplexing of up to 40 different SNPs per run, if properly designed [39]. The RT-PCR/ESI-MS is not limited to analysis of one SNP in an amplicon; it rather determines the base counts of the amplicon in a form of a signature. These signatures are then used for sample identification. Neither of these two technologies generates sequences as do other NGS platforms such as 454 GS-FLX system, Illumina, and Sequencing by Oligonucleotide Ligation and Detection (SOLiDTM) [39], [40]. The 454 GS-FLX system specifications states that it can sequence an average of 100 million DNA bases in a 7.5-h run, with an average read lengths of 250 bases (http://www.454.com). However, it does not allow the in-depth coverage and massive parallel sequencing provided by Illumina (http://www.illumina.com) or SOLiDTM (http://solid.appliedbiosystems.com) systems, respectively. Of note the latter two technologies have the limitation of providing only short sequence reads of 35–50 bases and a complete run takes ∼3–5 days [39], [40]. All of the above mentioned technologies are of high throughput nature, and in some applications two or more of them were combined for more efficiency and accuracy [39].

The RT-PCR/ESI-MS is based on the use of neural network analysis to compare newly generated BC signatures to those of viruses already in a reference database. It is important to highlight that final identification of any virus (e.g. strain name, subtype, host, etc…) depends on the information contained in this reference database. The assay generates BC signatures for the sample tested; however, the other virus identifiers are determined based on comparison to virus sequences (generated outside the RT-PCR/ESI-MS assay) and other virus attributes (such as host, clade, etc…), in the database. It is also important to note that for the RT-PCR/ESI-MS assay to provide the most accurate and up-to-date results, the reference database must be continuously updated with new virus sequences and relevant strain information. The present study has contributed to the enrichment of the database with the addition of the new viruses' BC signatures and genomic prints. Noteworthy, neural network-based analyses, such as the one employed in the RT-PCR/ESI-MS, are often biased by their database content. For instance the number of genomic prints and/or BC signatures as well as the source of these signatures, how well up-to-date are the references in the databases…etc, and gene sequencing combined phylogenetic analyses still provides the most complete answer among currently available influenza surveillance tools.

Given the high throughput nature of the RT-PCR/ESI-MS assay and its ability to differentiate between H1N1pdm and seasonal viruses, it has become a useful tool for monitoring genome reassortments in these viruses.

The Influenza kit in its current format is not a diagnostic tool; however there are ongoing efforts to explore the usefulness of the assay for clinical applications. Primers for amplification of the two genes encoding the surface antigens (HA and NA) are not included in the current kit. The HA and NA subtypes are only inferred from the results of the “core” genes analysis. Such conclusions may be accurate only with stable subtypes of influenza that are not involved in reassortment. Therefore inference of the NA and HA subtypes based on the genomic prints, limited to ‘core’ genes, should be done with great caution. Notable to the current Influenza kit is its failure to include the influenza A PB2 gene segment. As such, the current assay would not identify an emerging virus that contains a PB2 gene acquired through reassortment. Addition of primers targeting these genes could greatly improve the assay, even if this would require designing assays that are subtype specific and/or are aimed at addressing specific issues, such as monitoring the possibility of reassortment between the pandemic viruses and H5N1 viruses.

Of importance is the fact that the RT-PCR/ESI-MS can be used for testing clinical specimens directly and does not require virus culturing; and therefore can be time and cost effective.

The RT-PCR/ESI-MS assay demonstrated its usefulness in performing initial screening of a large number of H3N2 viruses and grouping them in accordance with their molecular signatures thus saving time and resources.

The RT-PCR/ESI-MS assay also demonstrated its ability to identify and distinguish seasonal H1N1 viruses of various genetic groups (clades). This information could be valuable in monitoring for reassortment between viruses from different genetic lineages within the same subtype. It is important to note that such intra-subtype reassortments have resulted in the emergence and limited spread of viruses dually resistant to both the NA inhibitor oseltamivir and the adamantanes [30], [31]. The possibility of reassortment between oseltamivir-resistant H1N1 seasonal and H1N1pdm viruses is an obvious concern.

Our data demonstrate the ability of the RT-PCR/ESI-MS to detect and identify dually resistant viruses of seasonal H1N1 that appeared as a result of reassortment between clade 2B (oseltamivir resistant) and clade 2C (adamantane resistant) lineages of seasonal H1N1 viruses. It is important to emphasize, however, that in some rare cases, occurrence of new mutations in the region targeted for M1 primers could change the BC signature and complicate discrimination between clades 2C and 2B.

In addition to monitoring the pandemic and seasonal influenza viruses, the RT-PCR/ESI-MS assay could be useful in identifying and differentiating LAIV from infections with wild type viruses. Furthermore, the assay has proven its capacity to detect mixed infections of different types/subtypes of viruses ([25], Deyde et al., 2009 unpublished data).

Our data demonstrate that the RT-PCR/ESI-MS assay is a valuable tool for detecting emerging viruses, for large scale screening for genome variants, and in monitoring for reassortment events with the caveat that additional targets (to include all eight influenza segments) should be added to the assay. Finally, the assay could be greatly improved by using two primer sets for each segment. This would reduce the influence of SNPs occurring in one of the targets on the same segment, and strengthen the identification capability of the influenza RT-PCR/ESI-MS assay.

## Materials and Methods

### Viruses

Clinical specimens (throat swabs, nasal swabs and washes, nasopharyngeal swabs, blood, sputum) and viral isolates submitted to CDC as part of the Global Influenza Surveillance Network activities from various regions of the world were used for RNA extraction and randomly selected for analysis using RT-PCR/ESI-MS assay. The identified viruses included H1N1pdm (n = 277), seasonal influenza H1N1 (n = 302), H3N2 (n = 171), as well as triple reassortant swine viruses (n =  7) isolated from humans. Reference viruses with full genome sequences available in the public domain were used for RT-PCR/ESI-MS assay results comparison and identification; and they included: A/South Dakota/06/2007 (seasonal H1N1, clade 2B), A/Colorado/UR06-0053/2007 (seasonal H1N1, clade 2C), A/Texas/57/2009 (seasonal H1N1-clades 2B and 2C reassortant virus), A/Brisbane/10/2007 (H3N2), A/Ohio/01/2007 (triple reassortant swine H1N1), A/Michigan/09/2007 (triple reassortant swine H1N2), and A/California/04/2009 (H1N1pdm virus). B/Florida/04/2006 was also used as a reference virus in case type B viruses were present among the samples tested, a consequence of virus mistyping or mixture. A/Ann Arbor/6/60 (H2N2) and B/Ann Arbor/1/66 viruses were used as reference viruses for LAIV strains.

Genomic prints of these viruses were determined based on their known genetic sequences and were used as references in the database.

### Ethics Statement

The Global Influenza Surveillance Network is public health surveillance, not human subjects' research. Therefore, evaluation of left-over surveillance specimens did not require IRB review or informed consent.

### Viral RNA isolation

RNAs were extracted using either MagNA Pure Compact RNA isolation kit, MagNA Pure LC total nucleic acid kit or MagNA Pure LC 2.0 RNA isolation kit (Roche, Indianapolis, IN). The viruses tested were initially characterized by rRT-PCR and/or hemagglutination inhibition (HI) tests at the Influenza Division, CDC.

### RT-PCR/ESI-MS assay

In brief, based upon analysis of multiple influenza sequence alignments, pan-influenza virus RT-PCR primer sets were developed to generate information-rich sequences as previously detailed [25]. An influenza assay (Cat #03N39-01, Abbott Molecular, Des Plaines, IL) was then designed, consisting of eight primer pairs, including one pan-influenza primer pair targeting the PB1 segment, five pan-influenza A primer pairs targeting NP, M1, PA and NS (NS1 and NS2 genes), and two pan-influenza B primer pairs targeting NP and PB2 gene segments. All primers used in this study had a thymine nucleotide at the 5′-end to minimize addition of non-templated adenosines during amplification using Taq polymerase [41]. The sensitivity of each RT-PCR primer pair was determined using known quantities of a synthetic calibrant RNA template as described previously [42].

One-step RT-PCR was performed as previously described [25]. Following amplification and product purification, molecular masses and base counts of amplicons were determined, using the T5000 platform, as previously described in detail [25], [42]–[45]. Semi-quantitative results of viral RNA genome copy numbers were obtained by comparing the peak heights with an internal PCR calibration standard present in every PCR well at 100 molecules [43].

### Limit of detection (LoD) studies on the H1N1pdm specimens

To determine the overall limit of detection of the RT-PCR/ESI-MS assay from nucleic acid extraction to data analysis, two different extraction methods with spikes of the H1N1pdm virus, A/California/04/2009, in two different matrices, nasal swab and nasal wash, were used to establish the LoD. Ten serial dilutions of the pandemic strain were prepared in the two matrices. RNA from each diluted sample was independently extracted using the Ambion MagMax® Viral RNA Isolation kit (Applied Biosystems/Ambion, Austin, TX) and using the Qiagen QIAamp® MinElute Virus Spin kit (Valencia, CA). Extracted RNA was added to the RT-PCR/ESI-MS assay kit and the plates were thermocycled on the Eppendorf Master cycler (Hauppauge, NY). The amplicons were then tested on the T5000 machine (Ibis/Abbott, Des Plaines, IL). The LoD was calculated to determine the lowest detectable concentration range of influenza virus at which ≥95% of all replicates across extraction methods tested positive. A positive result was defined as the detection of at least five of the six amplicons whose BC signatures match the H1N1pdm strain, resulting in the T5000's call of the pandemic virus.

## Supporting Information

Figure S1 RT-PCR/ESI-MS identified initial H1N1pdm strain as unusual virus with genome components of swine, human, and avian origin.
(2.09 MB TIF)

Figure S2 Limit of detection (LoD) studies on the H1N1pdm specimens.
(0.72 MB TIF)

Figure S3 Results of RT-PCR/ESI-MS analysis of an H1N1pdm LAIV case.
(0.40 MB TIF)

Table S1 Distribution of influenza A viruses analyzed in this study based on subtype and collection period.
(0.03 MB DOC)

Table S2 Analytical Limits of Detection (LoD).To assess and determine the limit of the detection of the RT-PCR/ESI-MS assay, analytical limits of detection studies (see Materials and Methods) were performed using the 2009 H1N1pdm strain. The results showed that the lowest detectable concentrations of influenza virus varied from 7.0×101 TCID50/ml to 2.11×102 TCID50/ml, depending on the matrix used: nasal swab or nasal wash (Table S2). It should be noted that these viral copy numbers determined by TCID50s may over look presence of defective interfering (DI) particles and therefore the lowest detectable number of molecules could be higher. Analysis of sensitivity of the assay based on viral copy numbers revealed that the LoD of the assay was 31 and 62 genome copies, from the nasal swab and nasal wash, respectively, using the Ambion MagMax®Viral RNA Isolation kit. The same analysis using the Qiagen QIAamp® MinElute Virus Spin kit showed the LoD of the assay was 125 and 62 viral copy numbers, from the from the nasal swab and nasal wash, respectively. Of note, the limit of detection here (the numbers) is considered only if all primers generated PCR products and BC signatures in the assay. Reference: Teresa Zembower, Varough Deyde, Larisa Gubareva, Alexander Klimov, Sudhir Penugonda, Kevin Kunstman, Maureen Bolon, David Dittman, Lawrence Blyn, David Ecker, Steven Wolinsky, Rangarajan Sampath. PCR/ESI-MS Approach for High-Throughput Identification of 2009 Pandemic Influenza A Viruses. 49th Interscience Conference on Antimicrobial Agents and Chemotherapy (ICAAC), San Francisco, California, September 12 - 15, 2009
(0.04 MB DOC)
