# Supplementary figures and images for: Genomic Signature-Based Identification of Influenza A Viruses Using RT-PCR/Electro-Spray Ionization Mass Spectrometry (ESI-MS) Technology
Source: PLoS One. 2010 Oct 12;5(10):e13293. doi: 10.1371/journal.pone.0013293 (PMC2953491; doi:10.1371/journal.pone.0013293)

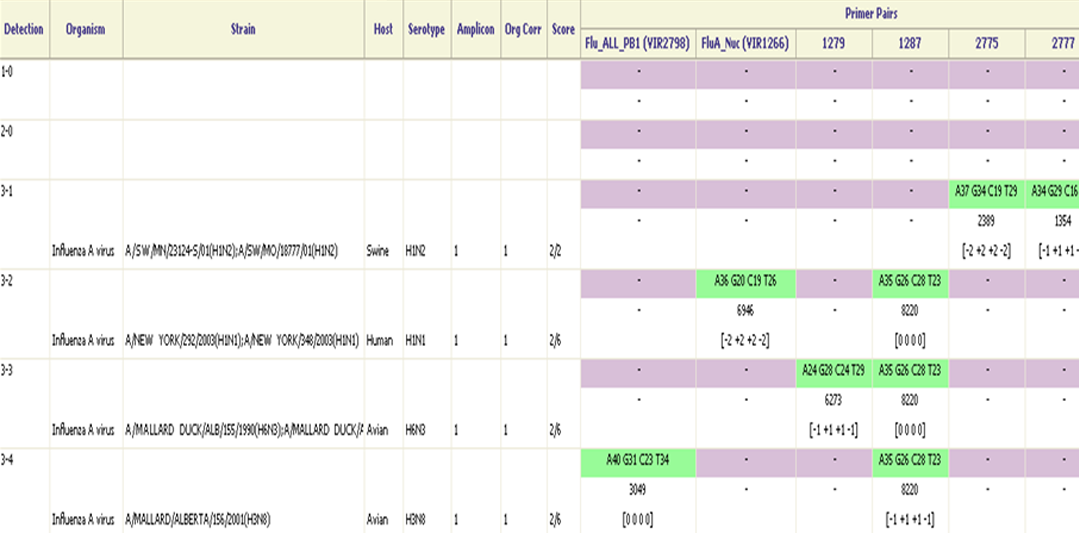

Supplement: Figure S1 — RT-PCR/ESI-MS identified initial H1N1pdm strain as unusual virus with genome components of swine, human, and avian origin. (2.09 MB TIF) [file pone.0013293.s001.tif]

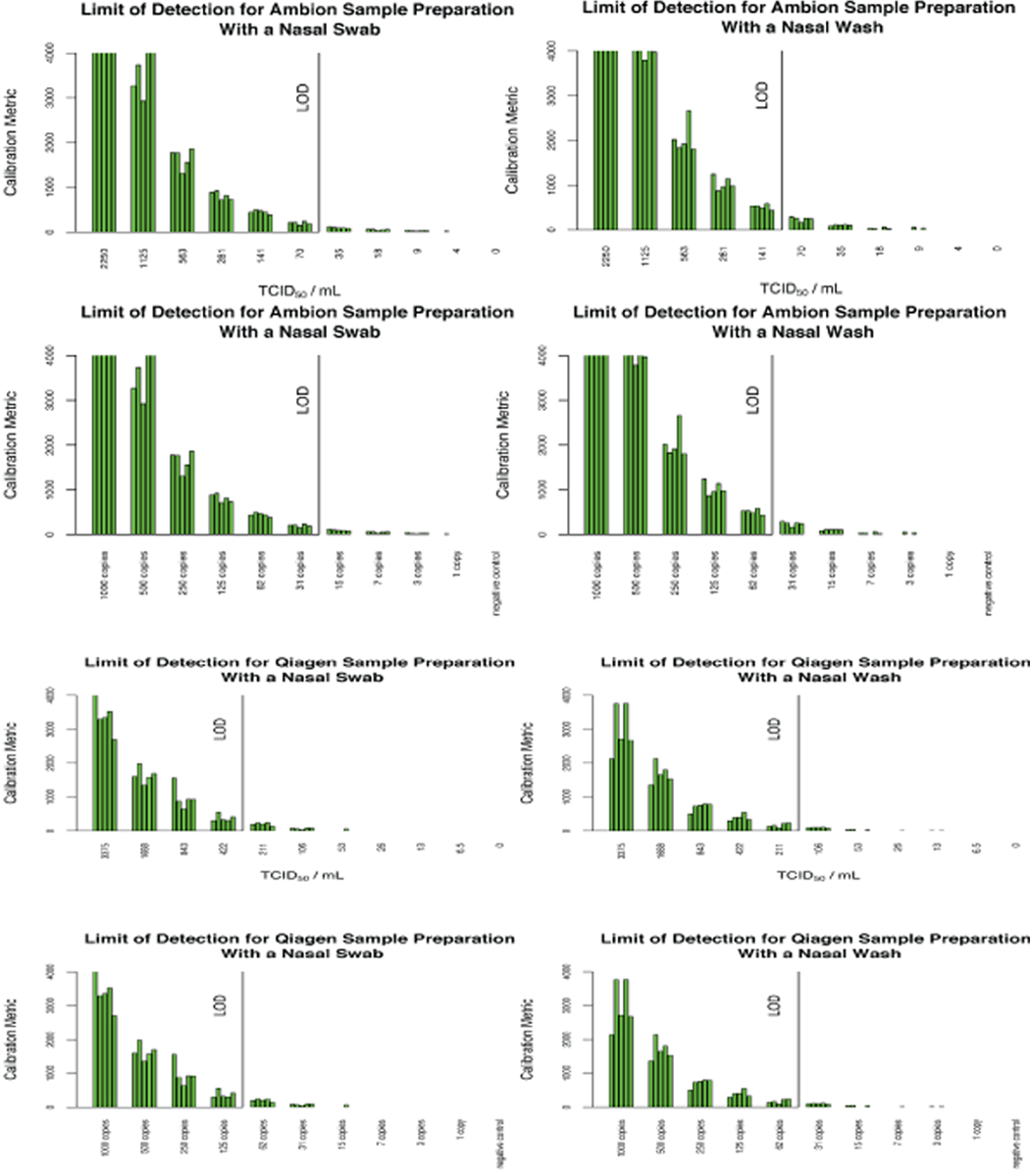

Supplement: Figure S2 — Limit of detection (LoD) studies on the H1N1pdm specimens. (0.72 MB TIF) [file pone.0013293.s002.tif]

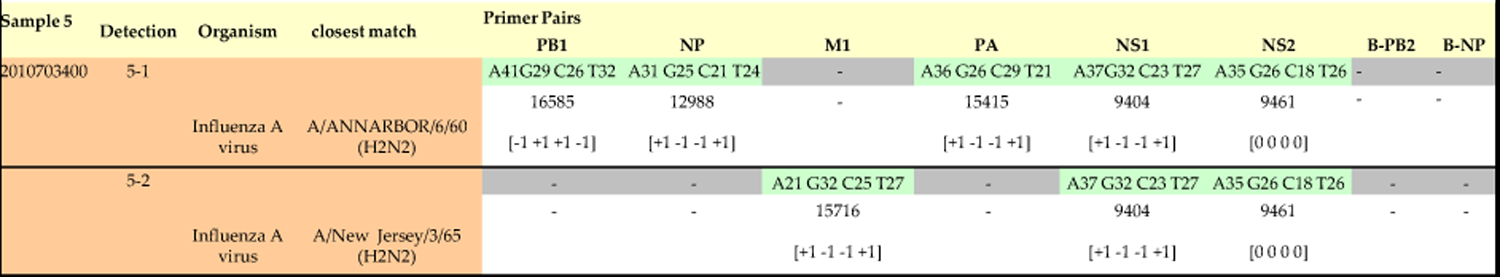

Supplement: Figure S3 — Results of RT-PCR/ESI-MS analysis of an H1N1pdm LAIV case. (0.40 MB TIF) [file pone.0013293.s003.tif]
