# Supplementary material for: Genomic Signature-Based Identification of Influenza A Viruses Using RT-PCR/Electro-Spray Ionization Mass Spectrometry (ESI-MS) Technology
Source: PLoS One. 2010 Oct 12;5(10):e13293. doi: 10.1371/journal.pone.0013293 (PMC2953491; doi:10.1371/journal.pone.0013293)
Supplement: Table S1 — Distribution of influenza A viruses analyzed in this study based on subtype and collection period. (0.03 MB DOC) [file pone.0013293.s004.doc]

**Table S1:** Distribution of influenza A viruses analyzed in this study based on subtype and collection period.

|  | 2006-07 | 2007-08 | 2008-09 | **Total** |
| --- | --- | --- | --- | --- |
| Seasonal: H1N1 | 130 | 74 | 98 | **302** |
| H3N2 | 76 | 57 | 38 | **171** |
| Swine triple reassortant | 5 | 1 | 1 | **7** |
| H1N1pdm | - | - | 277 | **277** |
| **Total** | **211** | **132** | **414** | **757** |
