# Supplementary material for: Genomic Signature-Based Identification of Influenza A Viruses Using RT-PCR/Electro-Spray Ionization Mass Spectrometry (ESI-MS) Technology
Source: PLoS One. 2010 Oct 12;5(10):e13293. doi: 10.1371/journal.pone.0013293 (PMC2953491; doi:10.1371/journal.pone.0013293)
Supplement: Table S2 — Analytical Limits of Detection (LoD).To assess and determine the limit of the detection of the RT-PCR/ESI-MS assay, analytical limits of detection studies (see Materials and Methods) were performed using the 2009 H1N1pdm strain. The results showed that the lowest detectable concentrations of influenza virus varied from 7.0×101 TCID50/ml to 2.11×10^2^ TCID50/ml, depending on the matrix used: nasal swab or nasal wash (Table S2). It should be noted that these viral copy numbers determined by TCID50s may over look presence of defective interfering (DI) particles and therefore the lowest detectable number of molecules could be higher. Analysis of sensitivity of the assay based on viral copy numbers revealed that the LoD of the assay was 31 and 62 genome copies, from the nasal swab and nasal wash, respectively, using the Ambion MagMax®Viral RNA Isolation kit. The same analysis using the Qiagen QIAamp® MinElute Virus Spin kit showed the LoD of the assay was 125 and 62 viral copy numbers, from the from the nasal swab and nasal wash, respectively. Of note, the limit of detection here (the numbers) is considered only if all primers generated PCR products and BC signatures in the assay. Reference: Teresa Zembower, Varough Deyde, Larisa Gubareva, Alexander Klimov, Sudhir Penugonda, Kevin Kunstman, Maureen Bolon, David Dittman, Lawrence Blyn, David Ecker, Steven Wolinsky, Rangarajan Sampath. PCR/ESI-MS Approach for High-Throughput Identification of 2009 Pandemic Influenza A Viruses. 49th Interscience Conference on Antimicrobial Agents and Chemotherapy (ICAAC), San Francisco, California, September 12 - 15, 2009 (0.04 MB DOC) [file pone.0013293.s005.doc]

**Table S2: Analytical Limits of Detection (LoD).**

| **Virus** | **Prep Kit** | **Matrix** | **LoD TCID_50_/mL** | **Results (Positive/Tests)** | **% Positive** |
| --- | --- | --- | --- | --- | --- |
| (A/California/04/2009) | Ambion | Nasal Wash | 1.41 x 10^2^ | 20/20 | 100% |
|  |  | Nasal Swab | 7.0 x 10^1^ | 20/20 | 100% |
|  | Qiagen | Nasal Wash | 2.11 x 10^2^ | 19/20 | 95% |
|  |  | Nasal Swab | 4.22 x 10^2^ | 20/20 | 100% |

To assess and determine the limit of the detection of the RT-PCR/ESI-MS assay, analytical limits of detection studies (see Materials and Methods) were performed using the 2009 H1N1pdm strain. The results showed that the lowest detectable concentrations of influenza virus varied from 7.0x10^1^ TCID_50_/ml to 2.11x10^2^ TCID_50_/ml, depending on the matrix used: nasal swab or nasal wash (Table S2). It should be noted that these viral copy numbers determined by TCID50s may over look presence of defective interfering **(**DI**)** particles and therefore the lowest detectable number of molecules could be higher. Analysis of sensitivity of the assay based on viral copy numbers revealed that the LoD of the assay was 31 and 62 genome copies, from the nasal swab and nasal wash, respectively, using the Ambion MagMax® Viral RNA Isolation kit. The same analysis using the Qiagen QIAamp® MinElute Virus Spin kit showed the LoD of the assay was 125 and 62 viral copy numbers, from the from the nasal swab and nasal wash, respectively. Of note, the limit of detection here (the numbers) is considered only if all primers generated PCR products and BC signatures in the assay.

**Reference:**

Teresa Zembower, Varough Deyde, Larisa Gubareva, Alexander Klimov, Sudhir Penugonda, Kevin Kunstman, Maureen Bolon, David Dittman, Lawrence Blyn, David Ecker, Steven Wolinsky, Rangarajan Sampath. PCR/ESI-MS Approach for High-Throughput Identification of 2009 Pandemic Influenza A Viruses. *49th Interscience Conference on Antimicrobial Agents and Chemotherapy (ICAAC)*, San Francisco, California, September 12 – 15, 2009
